# Gain control in olfactory receptor neurons and the detection of temporal fluctuations in odor concentration

**DOI:** 10.3389/fphys.2023.1158855

**Published:** 2023-07-12

**Authors:** Harald Tichy, Maria Hellwig

**Affiliations:** Department of Neuroscience and Developmental Biology, Faculty of Life Sciences University of Vienna, Vienna, Austria

**Keywords:** insect, odor plume tracking, zero wind, odor identity, concentration dynamics

## Abstract

The ability of the cockroach to locate an odor source in still air suggests that the temporal dynamic of odor concentration in the slowly expanding stationary plume alone is used to infer odor source distance and location. This contradicts with the well-established view that insects use the wind direction as the principle directional cue. This contribution highlights the evidence for, and likely functional relevance of, the capacity of the cockroach’s olfactory receptor neurons to detect and process—from one moment to the next—not only a succession of odor concentrations but also the rates at which concentration changes. This presents a challenge for the olfactory system because it must detect and encode the temporal concentration dynamic in a manner that simultaneously allows invariant odor recognition. The challenge is met by a parallel representation of odor identity and concentration changes in a dual pathway that starts from olfactory receptor neurons located in two morphologically distinct types of olfactory sensilla. Parallel processing uses two types of gain control that simultaneously allocate different weight to the instantaneous odor concentration and its rate of change. Robust gain control provides a stable sensitivity for the instantaneous concentration by filtering the information on fluctuations in the rate of change. Variable gain control, in turn, enhances sensitivity for the concentration rate according to variations in the duration of the fluctuation period. This efficiently represents the fluctuation of concentration changes in the environmental context in which such changes occur.

## Introduction

In the absence of wind and visual cues, cockroaches can perform pure odor-driven orientation to reach desired goal locations. Male cockroaches tracking a female pheromone odor plume in a laboratory wind tunnel quickly locate the pheromone source even when, after the start of the upwind run, the directional air flow is stopped (Willis et al., 2008). Some intrinsic features of the slowly expanding plume itself enable the cockroach’s up-tunnel orientation decision in still-air conditions. The underlying neural mechanisms are rather difficult to interpret because the instantaneous spatial and temporal distribution of odor concentration in windless or slowly moving air is unknown. A pure chemical orientation combined with the three-dimensional structure of odor plumes has been analyzed in detail for marine crustaceans in turbulent and non-turbulent airflow characteristics and in vegetated and smooth-bottomed habitats (Atema, 1996; Webster et al., 2001; Webster and Weissburg, 2001). Behavioral studies have shown that lobsters use a spatial gradient in the size and shape of the concentration pulse encounters to locate the odor source (Moore and Atema, 1988, 1991; Basil and Atema, 1994; Atema, 1996; Zettler and Atema, 1999). Electrophysiological recordings from the aesthetascs on the lateral filaments of the antennules of the spiny lobster, *Panulirus argus*, and the clawed lobster, *Homarus americanus*, revealed chemoreceptors that respond to a range of gradually increasing odor pulse concentrations (Marschall and Ache, 1989; Zettler and Atema, 1999; Derby et al., 2001). The authors proposed that lobsters detect and quantify the steepness of the onset-slopes or the rise time of the encountered odor pulses to identify the odor source direction and determine the diance from the source. The life styles and feeding ecologies of cockroaches differ considerably from those of lobsters. They prefer living near food sources in dark, enclosed places or indoors in food preparation areas, but as ground dwellers they can also use temporal odor pulse parameters to locate odor sources. To elucidate the neural mechanisms underlying such searching behavior, we tested the ability of the peripheral olfactory system to detect and quantify the steepness of the concentration pulses that cockroaches may use to infer odor source distance and location. The function of olfactory receptor neurons (ORNs) as pulse slope or pulse rise-time detectors depends on the extent to which the two parameters of changing concentration—the instantaneous odor concentration and its rate of change—influence the response.

## Material and methods

### Experimental animal

**FIGURE 1:**
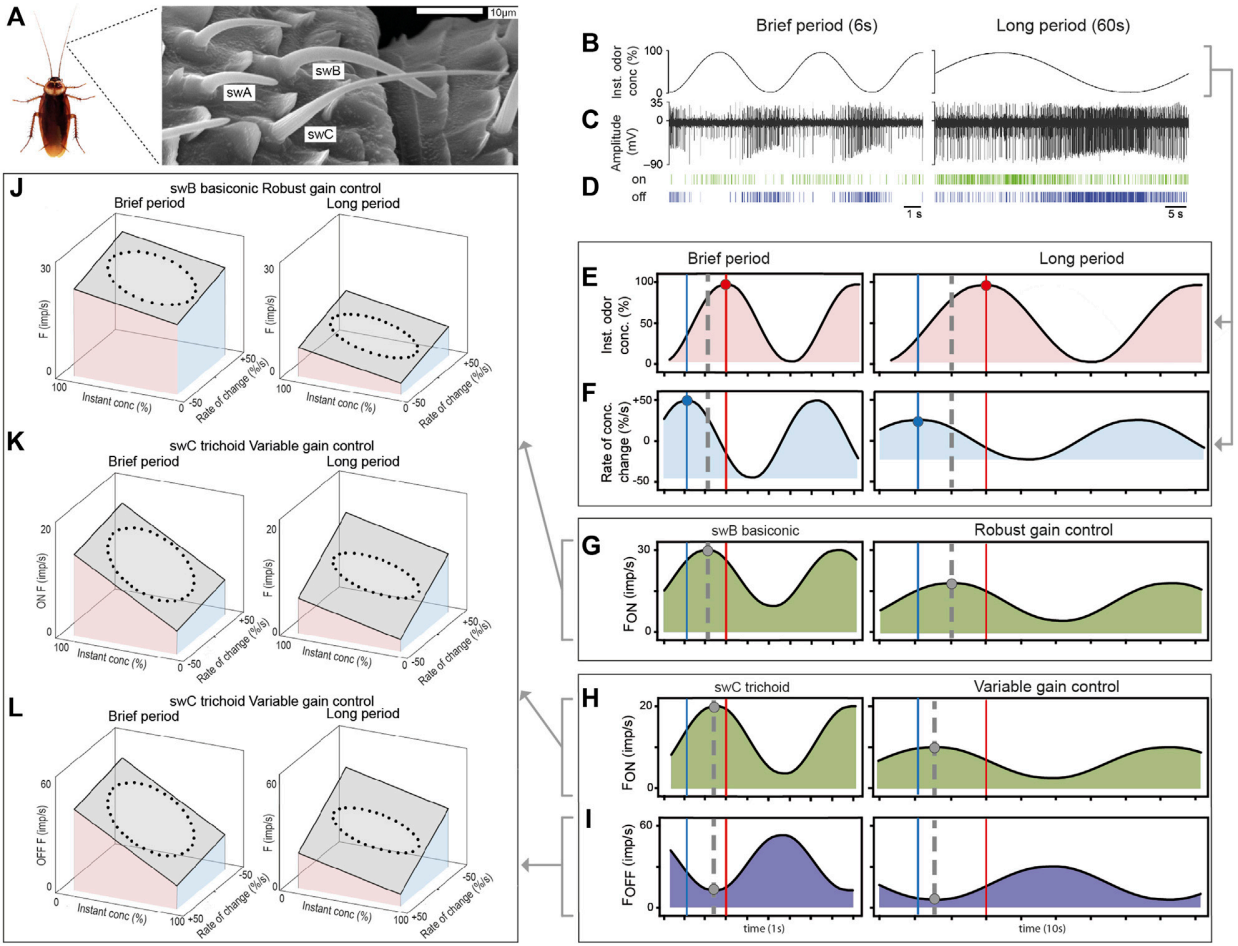
Olfactory receptor neurons on the cockroach antenna use gain control to encode the instantaneous odor concentration and its rate of change when the concentration of the odor of lemon oil slowly oscillates with different periods. **(A)** Scanning electron micrograph of different functional types of olfactory sensilla on the distal margin of a segment in the middle of the cockroach antenna. Single-walled type C (swC) basiconic sensilla contain antagonistically responding ON and OFF ORNs, single-walled type A and B trichoid sensilla (swA, swB) house ON ORNs (Burgstaller and Tichy, 2012; Tichy et al., 2020b). **(B)** Time course of oscillating odor concentration. **(C)** Simultaneously recorded impulses of ON and OFF ORNs located in swC trichoid sensilla. The OFF ORN displays larger impulse amplitudes than the ON ORN. **(D)** Raster plots illustrate the highly reproducible spiking patterns of the ON and OFF ORNs (Burgstaller and Tichy, 2012). **(E)** Time course of the instantaneous odor concentration. Red vertical lines: times of concentration maxima. **(F)** Time course of the rate of concentration change, leading the concentration function by a quarter period. Blue vertical lines: times of rate-of-change maxima. **(G)** Time course of the impulse frequency of an ON ORN of the swB basiconic sensillum performing robust gain control. The phase relationship between the response maxima (grey dashed line), the leading rate-of-change maxima and the lagging concentration maxima are invariant to the duration of the oscillation period (Tichy et al., 2020b). **(H)** Time course of the impulse frequency of an ON and OFF ORN of the swC trichoid sensillum performing variable gain control. The response maximum (grey dashed vertical line) of the ON ORN shifts during brief periods towards the concentration maximum (red vertical line), and during long periods, towards the rate-of-change maximum (blue vertical line). **(I)** According to the negative concentration coefficient of the OFF ORN, decreasing period duration shifts the response minimum (grey dashed vertical line) towards the concentration maximum, and a period increase towards the rate-of-change minimum (Burgstaller and Tichy, 2012). **(J)** Robust gain control of an ON ORN of the swB basiconic sensillum visualized by the best fitting regression planes for the observed impulse frequency, instantaneous concentration and rate of change. During both brief and long period durations, impulse frequency increases with rising instantaneous concentration and rising rate of change. The slopes for both variables are invariant to the period duration (for both regression planes: *R* ^2^ > 0.5, *p* < 0.001; Tichy et al., 2020b). **(K)** Variable gain control of an ON ORN of the swC trichoid sensilla found by the regression planes that best approximate the relationship between the sample data. The briefer the period duration and the faster the rate of change, the steeper is the slope of the instantaneous concentration and the flatter is the slope of the rate of change. Conversely, the longer the period and the slower the rate of change, the steeper is the slope of the rate of change and the flatter is the slope of the instantaneous concentration (for both regression planes: *R* ^2^ > 0.8, *p* < 0.001; Burgstaller and Tichy, 2012). **(L)** Variable gain control of an OFF ORN of the swC trichoid sensilla best represented by the fitted regression planes through the data points. During brief period durations and fast rates of change, the negative slope of instantaneous concentration becomes steeper and the negative slope of the rate of change become flatter. During long periods and slow rates of change, the negative slope of the rate of change becomes steeper and that of the instantaneous concentration flatter (*x* and *y* axis scales are reversed to K) (for both regression planes: *R* ^2^ > 0.8, *p* < 0.001; Burgstaller and Tichy, 2012).

The focus of our electrophysiological studies was on single-walled (sw) basiconic and trichoid sensilla located on the cockroach’s antenna which contain ORNs responsive to the odor of lemon oil (Burgstaller and Tichy, 2011; 2012; Tichy et al., 2020a). Schaller (1978) distinguished between short swA (length, 8–12 µm) and long swB basiconic sensilla (18–28 µm), and swC trichoid sensilla (30–40 µm) which are evenly tapered along their lengths, ending in a pointed tip (Figure 1A). The swA sensilla make up 8% of the total number of olfactory sensilla in the male cockroach, swB sensilla 54%, and swC sensilla 6% (Schaller, 1978; Altner et al., 1983).

### Dilution flow olfactometer

Slow and continuous changes in odor concentration at defined rates were provided by changing the flow rate ratios of an odor-loaded and a clean air stream. The mixing ratios of the two air streams were controlled by a pair of oppositely acting proportional valves. This enabled holding the total flow rate of the combined air stream constant at 1.5 m^−1^ (Tichy et al., 2020b). By delivering oscillating concentration changes from near zero to roughly 100% with periods of different durations, a given concentration change from one value to the next can be tested at different rates (Figure 1B). The rate of change of the up cycle reached values of ∼50% ⁄s during a 6-s period and ∼2% ⁄s during a 240-s period (Burgstaller and Tichy, 2012). Odor concentration was measured and monitored on-line by electronic flow meters, and the rate of change was calculated off-line. A critical feature of the novel olfactometer design is the risk of contamination when applying different odors which could be prevented by using different sets of odor tanks, tubes for the carrier stream, control valves, etc. This strategy quickly becomes unfeasible with an increasing number of odors, given space and cost constraints. Therefore, only a single odor was tested so far.

### Electrophysiology

Extracellular recordings of the activity of ORNs were obtained by tungsten-wire electrodes inserted into the base of the sensillum (Figure 1C). Spike identification and sorting were done off-line using the software Spike 2 (Figure 1D). Impulse frequency (F in imp⁄s) was calculated from running averages of three consecutive 0.2 s intervals (Burgstaller and Tichy, 2012; Tichy et al., 2020b). The schematic diagrams in Figure 1G–I are based on pooled response curves of 10 ORNs of different swB2 basiconic sensilla (Tichy et al., 2020b) and 16 ON and 16 OFF ORNs recorded as pairs from different swC trichoid sensilla (Burgstaller and Tichy, 2012).

### Statistical analyses

The phase of the oscillating impulse frequency was determined for different period durations by fitting each set of frequency data points with a sine wave curve (Figures 1G–I). Then the phase differences between the frequency maxima of ON ORNs and the frequency minima of the OFF ORNs (Figures 1G–I; grey dashed vertical lines), the maxima of the instantaneous concentration (Figure 1E; red vertical lines) and its rate of change (Figure 1F; blue vertical lines) were estimated. 3D plots were used to visualize the data and examine potential relationships among the impulse frequency, the instantaneous concentration and the rate of change. To this end, the values of the two independent variables of the odor stimulus (Figures 1E, F) and the dependent variable (Figures 1G–I) obtained at each point of time over an oscillation period were plotted with the rate of change along the *x* axis. The instantaneous concentration was plotted along the *y* axis, the impulse frequency along the *z* axis (Figures 1J, K). The resulting figure (known as a Lissajous figure) is elliptical because the three oscillating curves are out of phase. Least-squares multiple regression analyses were used to evaluate the effect of the two stimulus variables on the impulse frequency. The y slope of the regression plane represents the gain of response for the instantaneous concentration and the x slope the gain of response for the rate of change (Burgstaller and Tichy, 2012; Tichy et al., 2020a). A regression plane with a small (large) x slope is reached if the variable plotted on the *x* axis (the rate of change) has a slight (strong) effect on the gain of response for the variable plotted on the *y* axis (the instantaneous concentration). Similarly, a regression plane with a small (large) y slope indicates that the variable plotted on the *y* axis (the instantaneous concentration) has a slight (strong) effect on the gain of response for the variable plotted on the *x* axis (the rate of change).

## Results

### Different sensillum types encode different temporal features of the lemon oil odor

The ORNs of swA and swB basiconic sensilla simultaneously increased their activity with increasing odor concentration, though with different rates to different maxima (Tichy et al., 2020a), and the two ORNs of the swC trichoid sensilla responded antagonistically to the same fluctuations in odor concentration (Figure 1C). Increasing odor concentration raised the impulse frequency in the ON ORN and lowered it in the OFF ORN. Correspondingly, contrary effects were produced by decreasing odor concentration.

During oscillating concentration changes, the impulse frequency of the ORNs of both sensilla types oscillates regularly; the ratio of frequency oscillations to concentration oscillations was always 1:1. The frequency maxima of the ON ORNs of the basiconic sensilla were not in phase with the concentration maxima; they were intermediary, between the concentration maxima and the rate-of-change maxima (Figure 1G; grey dashed vertical lines). This phase relationship remained constant when the duration of the oscillation period varied, which means that the gain of response is invariant to the period duration. The ON and OFF ORNs of the trichoid sensilla also responded to both the instantaneous concentration and its rate of change, but their phase relationship varied with the period duration (Figures 1F, G; grey dashed vertical lines). With decreasing period duration, the frequency maxima of the ON ORNs and the frequency minima of the OFF ORNs shifted towards the concentration maxima (Figure 1H; red vertical line), which indicates a stronger dependence on the instantaneous concentration and a weaker dependence on the rate of change. Conversely, with increasing period duration, the frequency maxima shifted towards the rate-of-change maxima (Figure 1I; blue vertical line), indicating a stronger dependence on the rate of change and a weaker dependence on the instantaneous concentration.

### Robust gain control: ORNs of basiconic sensilla

To assess the extent to which the instantaneous concentration and the rate of change determine the ORNs activity during given oscillation periods, impulse frequency was plotted as a function of the two variables of the oscillating odor stimulus, and the relationship was approximated by the best fitting regression plane (Figure 1J). As indicated by the regression slopes, impulse frequency increases with rising concentration and increases more strongly the faster the concentration is rising through the higher values. Conversely, impulse frequency decreases with falling concentration and decreases more strongly the faster the concentration is falling through these same lower values. Thus, the effect of the instantaneous concentration is reinforced by the rate of concentration change. When the range of rates of change decreases by increasing the period duration, impulse frequency decreases but the reinforcing effect of the rate of change on the instantaneous concentration remains unchanged (Figure 1J). Robust gain control ensures a stable gain for the instantaneous concentration during fluctuating concentration changes, which facilitates encoding the odor identity (Tichy et al., 2020a).

### Variable gain control: ORNs of trichoid sensilla

The effect of the oscillation period on the phase relationships between the frequency maxima of the ON ORNs, the concentration maxima and the rate-of-change maxima is reflected in the changing slopes of the regression plane (Figure 1K). When the oscillation periods become briefer, the slope for the instantaneous concentration gradually becomes steeper and that for the rate of change flatter; and when the periods become longer, the slope for the rate of change gradually becomes steeper and that for the instantaneous concentration flatter. Thus, the gain for the instantaneous concentration is high during brief oscillation periods and higher still the briefer the period duration becomes. In contrast, the gain for the rate of change is high during long periods and higher still the longer the period lasts. Thus, during slow oscillations in odor concentration with long periods, the ORNs improve the gain for the rate of change at the expense of the gain for the instantaneous concentration. A high gain for the rate of change at slow rates enhances the ORN’s ability to detect creeping concentration changes, even if they progress in one direction. Contrarily, during rapid oscillations with brief periods, gain control trades an increased gain for the instantaneous concentration for a decreased gain for the rate of change. This prevents the ORNs from reaching saturation and losing information. Variable gain control provides high precision for slow rates, without reducing the detectable concentration range during fast rates or without expanding the impulse frequency range (Burgstaller and Tichy, 2012; Tichy and Hellwig, 2018).

The same relationship is evident between the frequency maximum of the OFF ORNs and the minima of both the rate of change and the instantaneous concentration (Figure 1L). The slopes of the regression plane for the OFF ORN are negative, as expected from an ORN whose discharge rate has a negative concentration coefficient. Detecting and processing concentration increments and decrements by a dual system of ORNs improve the efficiency of encoding temporal contrast information of fluctuating changes in concentration. Neither ORN simply signals changing odor concentration, but balances—from instant to instant—sensitivity according to the rate at which concentration changes.

## Discussion

The general orientation of male cockroaches toward a female pheromone source is mediated by the perception of the wind flow direction. In both laboratory and field odor plumes, the presence of wind flow polarizes olfactory-guided behavior in the up-stream direction (Willis and Avondet, 2005; Willis, 2008; Willis et al., 2008; Talley et al., 2023). However, video-recorded analysis of the walking tracks of male cockroaches in a laboratory wind tunnel demonstrate that when the males were half way to the source, stopping the wind flow has little effect on the success of locating the pheromone source. The cockroaches continued to track the now stationary plume, suggesting that they use only chemo-orientation mechanisms to follow the plume (Willis et al., 2008). Animals as diverse as lobsters perform true chemotaxis in that the spatial gradient in pulse size and shape guides orientation to the odor source (Moore and Atema, 1988; Basil and Atema, 1994; Zettler and Atema, 1999). The spatial distribution of onset slopes and the correlated pulse heights increase with decreasing distance to the odor source and provide the strongest spatial gradient pointing to the odor source (Moore and Atema, 1991; Atema, 1996). Electrophysiological recordings from chemoreceptors of the lateral antennules of the American lobster (Devine and Atema, 1982; Zettler and Atema, 1999) and from the ON and OFF ORNs in trichoid sensilla on the cockroach antennae revealed the existence of “pulse slope detectors” (Tichy and Hellwig, 2018).

**FIGURE 2:**
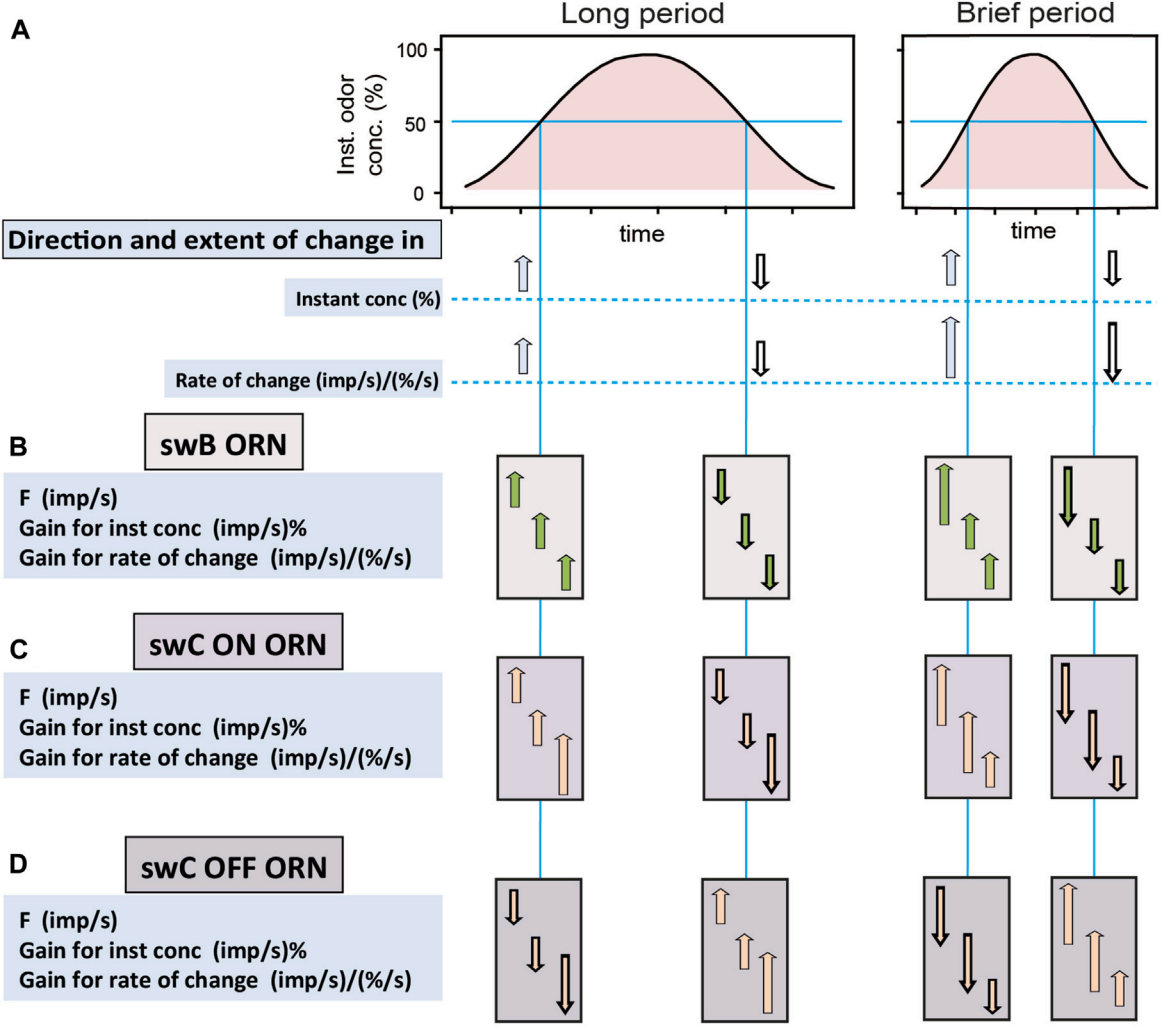
Characteristic responses of ORNs of basiconic and trichoid sensilla to constant-amplitude oscillating concentrations changes with long and brief periods (left and right side, respectively). **(A)** Time course of instantaneous odor concentration. Horizontal line indicates the concentration value chosen to compare the general response characteristics of the ORNs. **(B)** ON ORN of swB basiconic sensillum with robust gain control. **(C)** ON ORN of swC trichoid sensillum with variable gain control. **(D)** OFF ORN of swC trichoid sensillum with variable gain control. Arrows show direction and extent of change in value relative to the period between the changes. Read as follows, e.g., panel B, left side, onset slope of an oscillation with long period: “When odor concentration and the rate of change increase, then F of the ORN of the swB sensillum, the gain for instantaneous concentration and the rate of change rise”; and right side, onset slope of an oscillation with brief period: “When odor concentration and the rate of change increase more rapidly, then F of the ORN of the swB sensillum rises at greater rate, but the gain for instantaneous concentration and the rate of change do not rise stronger”. Gain control occurs for periods ranging from a few seconds to a few minutes.

While recent studies on the ON and OFF ORNs have clearly shown that the slower the rate of concentration change, the higher is their precision in differentiating small concentration changes (Tichy et al., 2016), their capacity to encode variations in temporally discontinuous concentration pulses was not examined in closer detail. The significance of intermittent odor signals in plume tracking and the role of the rate of filament or pulse encounter in anemotactic orientation for both flying and walking insects are well known (Willis and Baker, 1984; Murlis et al., 1992; Cardé and Willis, 2008; Lei et al., 2009; Cardè, 2021; Jayaram et al., 2022; Steele et al., 2023). Results from studies in moths and other insects indicate a nearly universal strategy for odor-source location that was framed as a surge upwind in the odor plume and a tendency toward cross-wind casting upon loss of the odor. Thus, the moment-to-moment contact with individual odor filament is a critical feature in tracking a turbulent odor plume and must be resolved with high fidelity by the insect’s olfactory system. Irrespective of the odor signal features used for localizing an odor source in turbulent wind flows or windless conditions, the sampling behavior is aimed principally to obtain spatial information on odor distribution and path direction from bilateral comparisons between their antennae or distant located receptive fields of the same antenna, or comparisons between two or more instants in time (Willis and Avondet, 2005; Willis et al., 2008; Lockey and Willis, 2015). Animals that orient by comparing bilateral odor concentration differences will turn toward higher concentrations. If the spatial gradient is represented by an increase in the rate of the pulse onset concentration, the pulse concentration alone will not lead to the source (Figure 2A). However, the ON and OFF ORNs on the cockroach’s antenna are specialized in detecting pulse onset slopes and could be particular useful for orientation along odor plumes in still air conditions. From the perspective of a cockroach, the problem of tracking a stationary odor plume is thus a temporal one, requiring detecting and quantifying the steepness or the rise time of the encountered odor pulses for guidance to their source. This contrasts to the iterative sequence of surging and casting behavior.

A characteristic feature of the ORNs of both basiconic and trichoid sensilla is that no impulse frequency refers simply to concentration or the rate of concentration change. Individual responses are ambiguous, not with regard to the direction of the concentration change but with regard to its extent. Each response can be elicited by various combinations of change in concentration and rate of change. The individual ORN therefore has limited ability to distinguish the two components of the odor stimulus. Perhaps cockroaches do not require precision in this regard. Rather, one may conceive that an increase in impulse frequency represents odor identity. In that case, the cue will be what odor elicits the increase in impulse frequency. Robust gain control acting in the ORNs of the basiconic sensilla is perfectly suited for this task. This is because it neglects variations in the rate of change due to variations in the duration of the fluctuation period. During oscillating odor concentration changes with varying periods (Figure 2A), however, different values of impulse frequency occur at the same instantaneous concentrations due to variations in the rate of change, but the gain for the two parameters remains constant (Figure 2B). The special feature of the swB basiconic sensilla is that they house two ORNs which respond both to changes in the concentration of lemon odor, but with different rate and strength. While their responses are different in absolute terms they maintain a constant relationship with one another as the concentration rate varies. The rank order of their stimulus response functions could serve as a criterion for coding odor identity that eliminates some of the ambiguity due to variations in both the concentration and the rate of change. Importantly, not only the rank order of excitation but also the ratios of responses (as a continuous variable) permits a concentration invariant odor code (Tichy et al., 2020a). Thus, encoding odor identity by relative activities of the two ORNs of swB basiconic sensilla provides a spatial activity pattern that may be constant over a range of concentrations and rates of change. As both ORNs are combined in the same basiconic sensillum, they have the same receptive field and perceive the same change in concentration at the same rate.

While robust gain control neglects variations in the rate of change due to variations in the duration of the fluctuation period, variable gain control emphasizes the detection of the rate of change on different temporal scales. Comparing the antagonistic responses of the ON and OFF ORNs in the trichoid sensilla may inform the cockroach as to when and in what direction concentration is changing (Figures 2C, D). Impulse frequency of both types of ORNs to a given concentration change tends to be higher generally, the faster the rate of change is (Figures 2C, D, compare left and right concentration wave). Although the same change in odor concentration activates the ON and OFF ORNs in opposite directions, it affects in a similar manner the gain for instantaneous concentration and the rate of change. Note that falling concentrations evoke stronger responses in the OFF ORN than rising concentrations in the ON ORN (Burgstaller and Tichy, 2011). A disparity for larger concentration changes could be an advantage for receiving information about falling concentration at the lateral edges of the odor plume than small rising concentrations within the plume. Clearly, strong responses of an ON ORN signal proximity to the source and most likely its direction. Weak responses will also do so, provided that a change in the cockroach’s course produces a stronger response in the OFF ORN. Strong responses of the OFF ORN indicate that concentration is falling. In this view, the cockroach uses the responses of the ON ORNs for distance and directional information and the responses of the OFF ORNs as alert or warning information.

The high gain of the ON and OFF ORNs to low rates of change would have an additional effect. Once the cockroach moves relative to the source, both ORNs will be confronted constantly with changing concentration. When the periods of the fluctuating odor concentration are long, a high gain for the rate of rising and falling odor concentrations will enhance the ORN’s ability to detect the fluctuating concentration (Figures 2C, D, left side). The cockroach will perceive the information on the concentration level at which the changes occur and can move to an area of higher concentration level closer to the odor source. Near the source, the periods of the fluctuating odor concentration will be brief (Figures 2C, D, right side). A high gain for the rate of change will achieve continually varying impulse frequency, and the faster concentration changes, the faster impulse frequency will vary. An advantage for representing concentration changes is provided only if an ORN has a limit on the gain for the rate of change. Without such a limit the ORN will always fluctuate and indicate concentration change. If the cockroach leaves the odor plume, the impulse frequency of the ON and OFF ORNs will become steady at some low rate of concentration change due to long fluctuation periods. The main information will be that the impulse frequency begins to change. A high gain for the rate of change will improve the ability to detect low rates of concentration changes. Because of the high gain for the rate of change, the cockroach will receive creeping changes in odor concentration, even if they persist in one direction. Gain control permits a high degree of precision at small rates when it counts most, without sacrificing the range of detection and without extending the measuring scale.

The temporal response characteristics of the ON and OFF ORNs are consistent with the idea that plume trackers walking on a smooth surface in a stationary or slowly expanding odor plume may use the change in shape and size of the encountered concentration pulses including their gradients in space and time to localize the odor source. Walking and in-flight plume tracking in turbulent flows, where odor concentration is patchily distributed resulting in temporally intermittent stimulation, requires measuring the timing of odor on and off and representing the intermittent plume as a spatiotemporal entity of the constituent odor filaments, but not filament concentration or its rate of changes (Martin et al., 2011; Steele et al., 2023; Talley et al., 2023). Wind tunnel studies suggest that two temporal features—odor intermittency and encounter frequency—can enhance the navigation of turbulent odor plumes in many moth species and *Drosophila*, which run or fly faster and straighter upwind when receiving odor hits at higher frequency than lower ones (Mafra-Neto and Cardè, 1994; Vickers and Baker, 1994; Keller and Weissburg, 2004; Jayaram et al., 2022). The high temporal resolution of ORNs is regulated intracellularly by their own olfactory receptor (OR), expressed at the dendritic membrane. The OR couples through a G-protein cascade to a cAMP-mediated second messenger pathway that gates an ion channel complex formed by the co-receptor Orco. The sensitivity of Orco for cAMP, and thus for the odor, is controlled by the degree of phosphorylation via PKC protein kinase C (Getahun et al., 2013; 2016; Wicher, 2018; Zufall and Domingos, 2018; Wicher and Miazzi, 2021). A lower quantity of odor molecules below the threshold of odor stimulation can significantly increase the sensitivity of OR for the odor, and if a second odor pulse encounters within a given time span, a response will be elicited. This upregulation of OR sensitivity could lead to assume that not only fewer molecules below the response threshold would amplify OR sensitivity, but also lower rates of concentration change such as shallow pulse slopes. If this is true, it seems reasonable to assume that the second-messenger pathway could control the gain of ON and OFF responses by tight balancing between high sensitivity for instantaneous concentration and the rate of concentration change due to the period duration. The polarity of the response gain is defined principally by the sign of the ON and OFF responses reflecting a polarity of transduction but not necessarily a polarity of the second messenger pathway. Selectively staining of ORNs by anti-PameORco antiserum (PameORco: *Periplaneta americana* ORco) suggests that the ON and OFF ORNs use different types of olfactory receptors (Tateishi et al., 2022). This finding indicates that the antagonistic ON and OFF responses are not the result of ephaptic coupling or non-synaptic inhibition as described for ORNs located in the same sensilla on the *Drosophila* antenna (Su et al., 2012), but represent true parallel pathways (Tichy and Hellwig, 2018).

Odorant binding proteins (OBPs) are widely considered to play a crucial role in the transport of hydrophobic odor molecules through the hydrophilic fluid inside the sensilla from the wall pores to ORs. OBPs form a specific complex with a given odor that interacts with ORs, leading to the initiation of the olfactory transduction cascade. A type of basiconic sensillum (ab8) on the *Drosophila* antenna contains a single highly expressed OBP, called Obp28a, which is not acting as carrier of volatile molecules (Larter et al., 2016; Sun et al., 2018; Xiao et al., 2019). Instead, the presence of Obp28a reduces the initial response to concentration pulses and prolongs the response after termination of a long pulse of high concentration. Response reduction will be achieved by binding some of the odor molecules to Obp28a and prolonged responses by releasing odor molecules from the OBP-odorant complex, which makes available either smaller or larger quantities for receptor binding. In both cases the OBP buffers the sensitivity of the system against rapid on and off changes in odor concentration. Could such a buffer effect of OBPs balance the gain of the ON and OFF ORN? During brief oscillations, OBPs should buffer against the rate of concentration change, and during long periods, against the level of the concentration change. Pore tubules provide an alternative to the carrier function of OBPs. They extend through the cuticular wall inward into the sensillum lumen and serve as a route for the odor molecules to reach the OR in the surface of the dendrites of the ORNs (Larter et al., 2016). The ON and OFF ORNs are located in single-walled trichoid sensilla with pore tubules and there has been no report regarding the expression of OBP (Schaller, 1978).

The pheromone-ORNs of the trichoid sensilla on the antenna of the male moth *Bombyx mori* emphasize the onset and offset of rapid concentration changes by functioning as flux detectors and not concentration measures (Kaissling, 1998). Flux detectors provide for a six orders of magnitude higher sensitivity (Rospars et al., 2000) by adsorbing and accumulating the odor molecules for longer time in the perireceptor space near the dendrites of the ORNs than concentration detectors. In order to indicate changes in flux, flux detectors prevent the perireceptor space to come into equilibrium with the external space but rather concentrates the molecules at onset and dilutes them at offset. Because odor molecules cannot leave the perireceptor space, they must be inactivated by pheromone-degrading enzymes to avoid overstimulation and maintain the ORN’s capacity to follow fast temporal changes in odor concentration (Rospars et al., 2000; Kaissling, 2009; Baker et al., 2012). In concentration detectors, the internal stimulus concentration at the ORN is in equilibrium with the external stimulus concentration. Stimulus molecules would be absorbed and desorbed within a few milliseconds so that there is no need for odor deactivation or odor degrading (Gu and Rospars, 2011).

Flux detectors adsorb the stimulus molecules depending on both the stimulus concentration within the external medium and the relative velocity of the flux detector and the airspeed (Kaissling, 1998; Rospars et al., 2000). The greater the airspeed, the greater is the flow rate (the air volume flowing across an area per unit of time) of the odor-loaded air stream. An increase in the flow rate of an air volume at constant concentration does not result in any change in the number of molecules per unit volume (the ratio between molecule number and air volume), but does increase the absolute number of molecules delivered per unit time. Thus flux detectors reveal the measure of concentration in molecules per area and per time. The response to equal rates of concentration change would increase with increasing flow rate level due to the increasing absolute number of odor molecules arriving at the sensillum. Flux detectors are not primarily working as sensors for the rate with which concentration changes, but as sensors of stimulus presence with binary, on and off response (Rospars et al., 2000). In concentration detectors, the odor concentration in the perireceptor space is in equilibrium with the external concentration. The molecules can move freely and instantly between the two spaces. Concentration detectors are able to detect changes in the odor concentration regardless of the air volume size, the absolute number of molecules involved in the concentration change, the rate of arrival at the antenna or the rate of air flow. While ORNs were in general regarded as flux detectors (Kaissling, 1998), it took some time to then provide experimental evidence of true concentration detectors. This was accomplished in basiconic sensilla of *Drosophila* and was exemplary in showing that the responses of ORNs to rapid, pulse-like concentration changes are invariant to variations in the pulse flow rate (Zhou and Wilson, 2012). Thus, pulse concentration could be used by *Drosophila* to track along wind-borne odor plumes to their source. In a recent study we have shown that changing the level of the flow rate has no effect on the responses of the ON and OFF ORN responses to oscillating changes in odor concentration (Hellwig et al., 2019). Furthermore, the gain of both ORNs for the concentration rate is robust against the air flow velocity. This allows the instantaneous analysis of the rate of concentration change for both directions of change by one or the other ORN. Therefore, the ON and OFF ORNs are optimized to encode concentration increments and decrements in an odor plume and function as “concentration slope detectors”.

In conclusion, our findings suggest that key aspects of the odor stimulus are extracted and processed separately in two parallel systems of ORNs located in morphologically different types of sensilla on the cockroach’s antenna. The questions now are what mechanisms cause the two types of gain control and how does the brain determine what information is suitable at any given moment to guide the cockroach to the location of the odor source.

## Data Availability

The raw data supporting the conclusion of this article will be made available by the authors, without undue reservation.
